# A short large-calibre intrapelvic arterial bridge between the superior and inferior gluteal arteries: an in vivo CT angiographic observation

**DOI:** 10.1007/s00276-026-04029-z

**Published:** 2026-10-12

**Authors:** Mugurel Constantin Rusu, Sorin Hostiuc, Adelina Maria Jianu

**Affiliations:** 1 Division of Anatomy, Faculty of Dentistry, Carol Davila University of Medicine and Pharmacy, 050474 Bucharest, Romania; 2 Department of Legal Medicine and Bioethics, Carol Davila University of Medicine and Pharmacy, 042122 Bucharest, Romania; 3 Department of Anatomy and Embryology, Faculty of Medicine, “Victor Babeș” University of Medicine and Pharmacy, 300041 Timișoara, Romania; 4 Municipal Emergency Clinical Hospital of Timișoara, 300041 Timișoara, Romania

**Keywords:** Superior gluteal artery, Inferior gluteal artery, Internal iliac artery, Arterial anastomosis, CT angiography, Anatomical variation

## Abstract

**Purpose:**

To describe a short, large-calibre intrapelvic arterial bridge between independently established superior and inferior gluteal arteries (SGA and IGA) and to distinguish it from a common gluteal trunk.

**Methods:**

A pelvic computed tomography angiography (CTA) examination from a 40-year-old man was retrospectively analysed from an anonymised archive using multiplanar reformations, maximum-intensity projections, and three-dimensional vascular reconstructions. Linear and calibre measurements were obtained with workstation callipers and verified by independent review.

**Results:**

On the right, the internal iliac artery divided after 34 mm into anterior and posterior trunks. The anterior trunk gave the obturator and internal pudendal arteries and continued as the IGA (2.6 mm); the posterior trunk gave sacral arteries and continued as the SGA (4.0 mm). The SGA and IGA were connected 44.2 mm distal to the internal iliac bifurcation by a 1.8-mm-long, 3.5-mm-calibre intrapelvic bridge. The SGA exited suprapiriformly and the IGA infrapiriformly. No corresponding bridge was present on the left.

**Conclusion:**

The configuration differs from an Adachi Type II common SGA–IGA trunk because the two gluteal arteries are already anatomically separate before the communicating segment. Strict assignment to an Adachi type is not possible from this CTA alone because the umbilical continuation of the hypogastric axis is not demonstrated. Previous cadaveric studies documented SGA–IGA communications, whereas the present observation provides individual in vivo CTA topography and morphometry of a very short, large-calibre bridge.

## Introduction

The superior and inferior gluteal arteries (SGA and IGA) are major parietal branches of the internal iliac artery (IIA). Classical descriptions emphasise their origins and common trunks. Adachi classified the relationships of the SGA, IGA and internal pudendal artery (IPA) with reference to the A. hypogastrica–Lig. umbilicale laterale arterial axis into five principal patterns: Type I, separate SGA with a common IGA–IPA trunk; Type II, common SGA–IGA trunk with a separate IPA; Type III, all three arteries separate; Type IV, all three from a common trunk; and Type V, common SGA–IPA trunk with a separate IGA [1]. Because the umbilical continuation of this axis cannot be defined on the present CT angiography (CTA), strict assignment of the case to an Adachi type is limited. The relevant morphological distinction is nevertheless clear: the observed SGA and IGA are already separate before the short intrapelvic communication and therefore do not form a Type II common gluteal trunk.

Direct SGA–IGA communication has, however, been documented. Kuzmenko [9] photographed intrapelvic SGA–IGA anastomoses in cadaveric specimens, and Kuzmenko and Shkvarko (2020) later quantified these communications [10]. Other reports describe morphologically distinct connections, including a complete intrapelvic arterial ring [8] and a deep communication between gluteal vessels during a flap dissection [7]. This report characterises, by CTA, a unilateral, extremely short, large-calibre SGA–IGA bridge with complete bilateral arterial context. Recent CTA studies characterised the origins and branching patterns of the IGA and SGA but did not identify a comparable main-stem SGA–IGA bridge [5, 6].

## Materials and methods

### Study design and ethical approval

This was a retrospective anatomical analysis of a clinically acquired pelvic CT angiographic data set from an anonymised imaging archive. No additional imaging, contrast administration, radiation exposure, or intervention was performed for this study. The study was conducted in accordance with the Declaration of Helsinki. Approval was granted by the Municipal Emergency Clinical Hospital of Timișoara, Romania (approval no. E-4247/15.10.2025). The study was conducted in accordance with current legislation, patient rights, and data-protection regulations, including GDPR Regulation no. 679/2016. We used the same ethical framework for the retrospective analysis of anonymised CT angiographic material as in our previously published radiological anatomy work [12].

### Image analysis and measurements

The pelvic arterial tree was evaluated bilaterally on a Horos DICOM workstation (Horos v3.3.6) using axial, coronal and sagittal multiplanar reformations, maximum-intensity projections, and three-dimensional vascular reconstructions. The common, external, and internal iliac arteries and the major parietal branches of the internal iliac artery were followed continuously on source and reconstructed images to determine their origins, branching sequence, and relationship to the piriformis and the greater sciatic foramen. Particular attention was paid to whether the SGA and IGA shared a proximal trunk or were independently established before the communicating vessel was identified.

Linear and calibre measurements were obtained directly from the DICOM data using the workstation calliper tool. The length and calibre of the communicating segment, the calibres of the SGA and IGA, the length of the right IIA before its division, and the distance from the IIA bifurcation to the SGA–IGA bridge were recorded. Measurements were repeated in separate sessions, and final values were established after independent review and consensus between two senior researchers. We analysed the contralateral arterial tree using the same approach for internal anatomical comparison. Terminology follows the Terminologia Anatomica where applicable; “anterior” and “posterior trunks” of the IIA are retained as conventional descriptive terms.

## Results

Pelvic CTA of a 40-year-old man demonstrated asymmetric bilateral branching of the common and internal iliac arterial systems.

### Right side

**Fig. 1:**
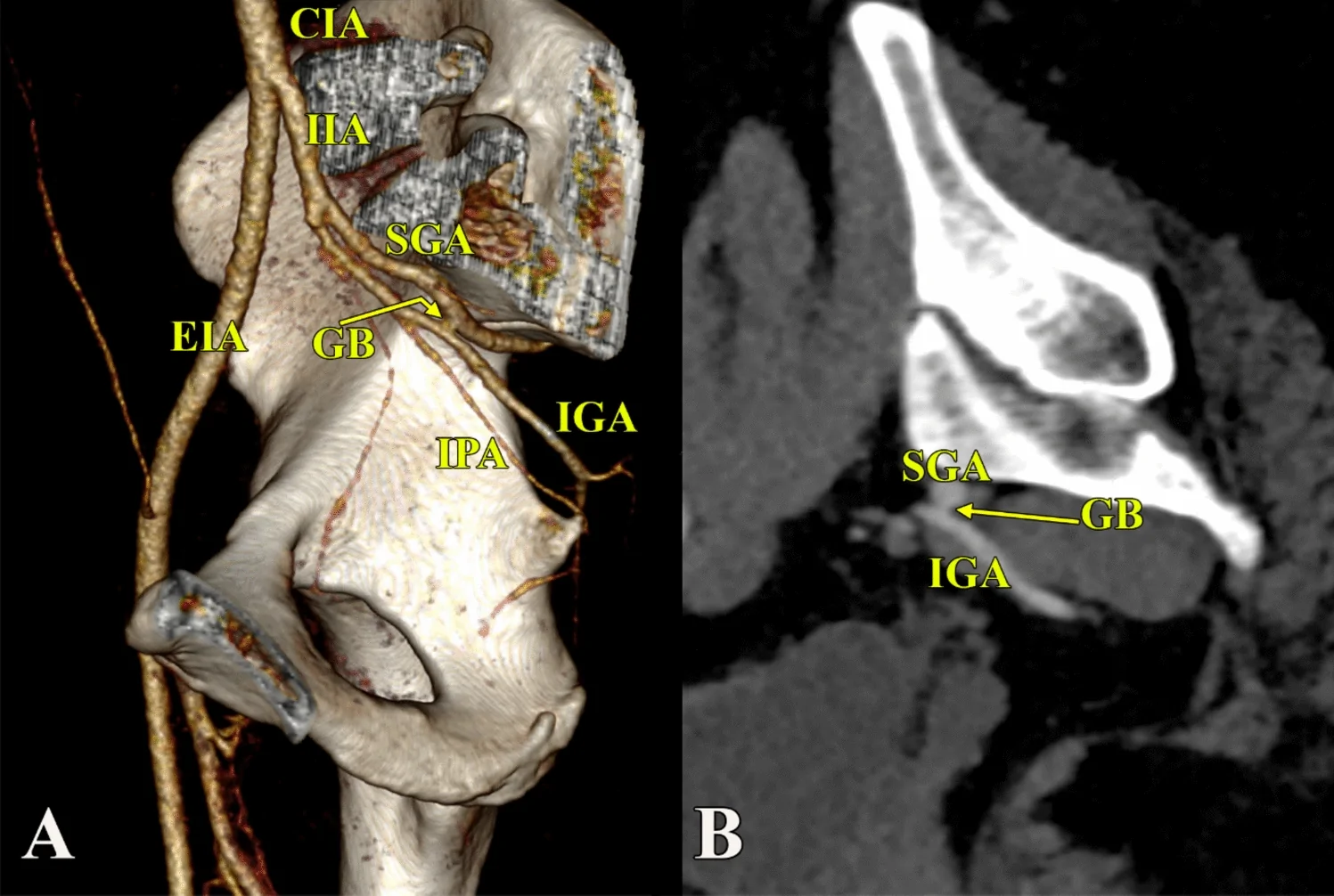
Right-side evidence of the bridge between the gluteal arteries. **A** Three-dimensional volume rendering, antero-medial view. **B** Sagittal slice through the gluteal arterial bridge. CIA: common iliac artery; EIA: external iliac artery; IIA: internal iliac artery; SGA: superior gluteal artery; IGA: inferior gluteal artery; IPA: internal pudendal artery; GB: gluteal arterial bridge

On the right side (Fig. 1), the common iliac artery (CIA) bifurcated into the external iliac artery (EIA) and IIA at the upper third of the L5 vertebral body. The IIA measured 3.4 cm before dividing into anterior and posterior trunks at the superior margin of the sacrum. The iliolumbar artery arose directly from the CIA. The anterior trunk gave rise to the obturator artery (OA) and IPA, then continued as the IGA, which measured 0.26 cm in calibre. The IGA coursed over the piriformis and passed through the infrapiriform space. The middle rectal artery arose from the IGA 1.15 cm proximal to the ischial spine. The posterior trunk gave off sacral arteries, then continued as the SGA, which measured 0.40 cm in calibre and passed through the suprapiriform space.

At 4.42 cm distal to the main bifurcation of the IIA, the SGA and IGA were connected by a short intrapelvic arterial bridge measuring 0.18 cm in length and 0.35 cm in calibre (Fig. 1). The bridge was therefore wider than it was long (length-to-calibre ratio, approximately 0.51); its calibre was approximately 88% of that of the SGA and 135% of that of the IGA.

### Left side

**Fig. 2:**
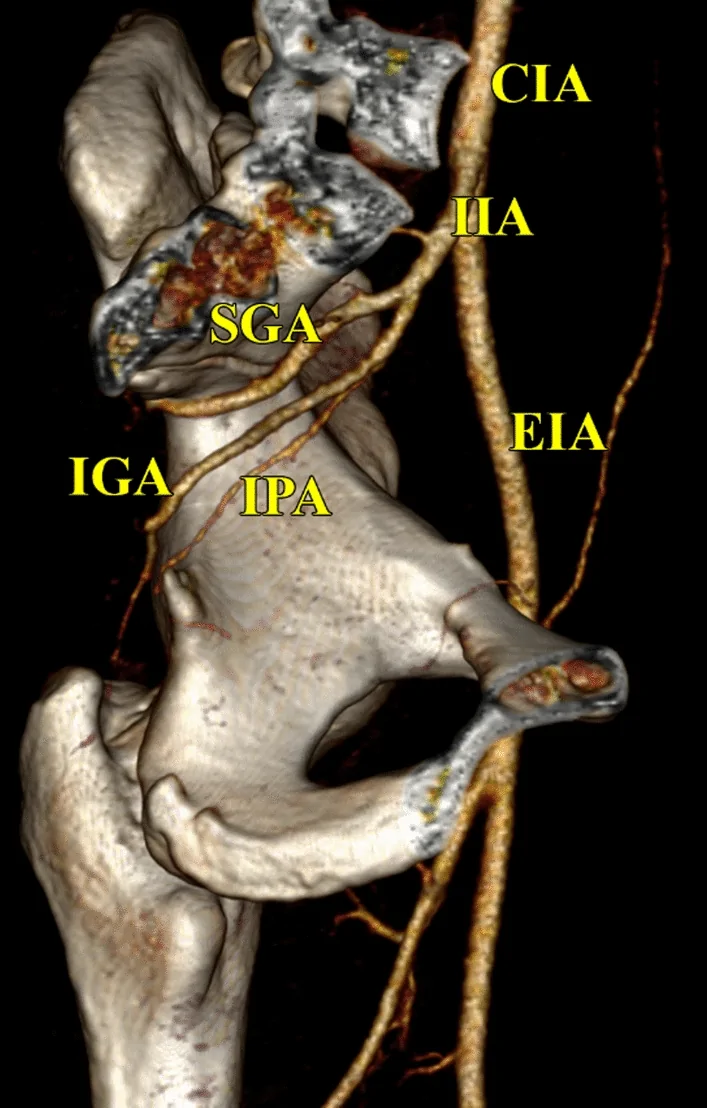
Left internal iliac arterial tree. Unbridged gluteal arteries. Three-dimensional volume rendering. Medial view. CIA: common iliac artery; EIA: external iliac artery; IIA: internal iliac artery; SGA: superior gluteal artery; IGA: inferior gluteal artery; IPA: internal pudendal artery

The CIA bifurcated at the lower third of L5, and the IIA divided into anterior and posterior trunks at S2. The iliolumbar artery again arose from the CIA. The anterior trunk gave the IPA and IGA, whereas the OA shared a common origin with the inferior epigastric artery from the EIA. The IGA gave the middle rectal artery. The posterior trunk gave sacral arteries and continued as the SGA. No direct intrapelvic SGA–IGA communication was identified (Fig. 2).

## Discussion

The present configuration should not be labelled an Adachi Type II pattern. In Adachi’s original framework, classification depended on the relationships of the SGA, IGA and IPA to the A. hypogastrica–Lig. umbilicale laterale stem; Type II specifically describes a common Truncus glutaealis that divides into SGA and IGA [1]. Because the umbilical continuation is not depicted on the present CTA, strict assignment to Type III would exceed the information available. Morphologically, however, the SGA and IGA are already separate before the short communicating bridge, which distinguishes the finding from a common gluteal trunk. The present case is therefore described directly by its observed topology rather than by implying a temporal or developmental sequence.

Kuzmenko provided the most direct anatomical precedent. Kuzmenko [9] reported an intrapelvic SGA–IGA anastomosis in two of three published cadaveric figures, one in a 59-year-old man and one in an 82-year-old woman. However, individual length and diameter were not reported for those specific communications; morphometry was pooled across all IGA intrapelvic anastomoses by sex and side. Kuzmenko and Shkvarko [10] found direct SGA–IGA communications in 7.5% of male right hemipelves, 7.5% of male left hemipelves, 2.8% of female right hemipelves and 7.3% of female left hemipelves. Again, mean length and diameter were reported for all SGA intrapelvic collaterals combined rather than for the SGA–IGA subset. The two studies came from the same institution and used closely similar bilateral unfixed-cadaver injection/dissection protocols; because cohort overlap was not reported, their denominators should not be pooled.

Other literature uses SGA–IGA communication in different anatomical senses (Table 1). Honma et al. reported a complete intrapelvic arterial ring connecting the gluteal arteries [8]. Their configuration formed a closed arterial loop/fenestration, whereas the present CTA demonstrates a single, very short communicating segment between otherwise separate SGA and IGA stems. Honma et al. proposed a developmental interpretation for their ring, but that interpretation was inferred from adult morphology rather than demonstrated by serial embryological observations. Georgantopoulou et al. illustrated a rare communication deep to piriformis during gluteal flap dissection [7], a location best regarded as deep gluteal/submuscular rather than a demonstrated main-stem intrapelvic bridge. Endovascular work has also treated preserved SGA–IGA communication as an important collateral pathway during IIA embolisation [4], supporting its functional relevance without defining the precise morphology of the communicating channel.

**Table 1:** Published descriptions relevant to SGA–IGA communication and comparison with the present case

Source	Setting	SGA–IGA relationship	Relation to the present case
Adachi [1]	Cadaveric classification	Type II common SGA–IGA trunk; Type III separate SGA, IGA and IPA	Classification depends on the hypogastric–umbilical axis; the present CTA cannot be strictly typed, and no discrete communicating-bridge category is defined
Honma et al. [8]	Single cadaveric case	Complete intrapelvic SGA–IGA arterial ring	Closed intrapelvic arterial ring/fenestration rather than a single bridge; developmental interpretation was hypothesis-based
Georgantopoulou et al. [7]	Fresh-cadaver flap study	Rare communication deep to piriformis	Direct communication, but deep gluteal/submuscular context
Kuzmenko [9]	Cadaveric injection/dissection	Two photographed intrapelvic SGA–IGA anastomoses	Direct anatomical precedent; no individual bridge morphometry
Kuzmenko and Shkvarko [10]	Cadaveric injection/dissection	SGA–IGA communication in 2.8–7.5% by sex/side	Best frequency evidence; no individual SGA–IGA morphometry
Fujioka et al. [4]	Endovascular/angiographic	Preserved SGA–IGA communication used as collateral pathway	Functional relevance; precise bridge anatomy not defined
Present case	In vivo CTA	Single 1.8-mm-long, 3.5-mm-calibre bridge between separate SGA and IGA	Individual bilateral topography and morphometry

The calibre of the present bridge is noteworthy. At 3.5 mm, it approached the calibre of the SGA (4.0 mm) and exceeded that of the IGA (2.6 mm), while its length was only 1.8 mm. This geometry supports the descriptive term “arterial bridge” or “communicating segment” rather than a long collateral branch. Static CTA cannot determine the direction or magnitude of flow, so the term “anastomosis” here remains morphological rather than haemodynamic.

The clinical implication is the possibility of alternative filling between arterial territories that may otherwise appear to arise from different IIA trunks. During selective embolisation or ligation, a large intergluteal connection could maintain distal perfusion or permit persistent bleeding beyond an occlusion. Fujioka et al. (2021) found that preservation of SGA–IGA communication was associated with better gluteal oxygenation after IIA embolisation [4]. The present bridge provides a clear anatomical substrate for such collateral continuity.

Direct embryological evidence for formation of an SGA–IGA bridge is unavailable. Honma et al.’s interpretation can, however, be placed within a broader developmental model of arterial remodelling. Rodríguez-Niedenführ et al. [11], studying 112 staged human embryos, showed in the upper limb that an initial capillary plexus is remodelled by selective enlargement and differentiation of channels, providing a mechanism by which variant arterial pathways can persist. Classic lower-limb studies likewise describe changing axial/sciatic and femoral arterial systems during development [2, 13]. By analogy, the present bridge may represent persistence or enlargement of an interarterial channel within the developing pelvic arterial network. This should be regarded as a plausible developmental hypothesis rather than a demonstrated mechanism for the present case.

The literature does not support claiming the first existence of SGA–IGA communication. The contribution of the present case is the combination of complete in vivo CTA context, unequivocally separate proximal SGA and IGA, exact localisation relative to the IIA bifurcation and piriformis exits, individual bridge dimensions, and contralateral comparison. Among the anatomical series reviewed, the Kuzmenko publications are the only studies specifically centred on simple intrapelvic gluteal arterial collateral branches [9, 10]. Dubreuil-Chambardel [3] provides detailed pelvic arterial grouping/origin variants, while the developmental works cited above address arterial remodelling. No additional report was identified with the same combination of a single short parent-stem SGA–IGA bridge, in vivo CTA demonstration, individual morphometry, and contralateral comparison. Neither Adachi’s original scheme nor the later Bergman-derived synthesis represents such an intrapelvic bridge as a discrete branching type [1, 14].

## Conclusion

This unilateral arterial bridge reconnecting independently arising SGA and IGA stems is distinct from a common gluteal trunk. Its extremely short course and large calibre are unusual, and CTA permits individual morphometric and bilateral topographic characterisation that is absent from the principal cadaveric series. This finding may be relevant to interpreting pelvic angiography and planning selective embolisation or ligation of gluteal arterial branches.

## Data Availability

The imaging data analysed in this study are not publicly available because they contain potentially identifiable clinical information. Anonymised data supporting the findings of this study may be made available by the corresponding author upon reasonable request, subject to applicable ethical and data-protection requirements.

## References

[1] Adachi B (1928) Das Arteriensystem der Japaner. Band II. Verlag der Kaiserlich-Japanischen Universität zu Kyoto, Kyoto, pp 97–110

[2] Cazenave-Mahé JP, Ducasse P, Videau J (1980) Embryologie des gros troncs artériels du membre pelvien de l’homme. Anat Clin 2:351–359. 10.1007/BF01558625

[3] Dubreuil-Chambardel L (1925) Traité des variations du système artériel. Variations des artères du pelvis et du membre inférieur. Masson et Cie, Paris, pp 46–56

[4] Fujioka S, Kitamura T, Mishima T, Nakajima R, Tamura Y, Horikoshi R, Araki H, Yakuwa K, Tomoyasu T, Okamura T, Miyamoto T, Torii S, Miyaji K (2021) Gluteal blood flow monitoring in endovascular aneurysm repair with internal iliac artery embolization. Circ J 85:345–350. 10.1253/circj.CJ-20-1002 33597321

[5] Gabryszuk K, Bonczar M, Ostrowski P, Gliwa J, Yika ADC, Iskra T, Klosinski M, Wojciechowski W, Walocha J, Koziej M (2024) The inferior gluteal artery anatomy: a detailed analysis with implications for plastic and reconstructive surgery. Folia Morphol (Warsz) 83:53–65. 10.5603/FM.a2023.0029 37144850

[6] Gabryszuk K, Gliwa J, Dziedzic M, Del Carmen YA, Ostrowski P, Bonczar M, Klosinski M, Wojciechowski W, Walocha J, Koziej M (2024) The superior gluteal artery and the posterior division of the internal iliac artery: an analysis of their complete anatomy. Folia Morphol (Warsz) 83:604–614. 10.5603/fm.96695 37957934

[7] Georgantopoulou A, Papadodima S, Vlachodimitropoulos D, Goutas N, Spiliopoulou C, Papadopoulos O (2014) The microvascular anatomy of superior and inferior gluteal artery perforator (SGAP and IGAP) flaps: a fresh cadaveric study and clinical implications. Aesthetic Plast Surg 38:1156–1163. 10.1007/s00266-014-0398-z 25209531

[8] Honma S, Aimi Y, Kudo M (2013) Ring formation of the internal iliac artery. Surg Radiol Anat 35:169–171. 10.1007/s00276-012-1020-1 22968634

[9] Kuzmenko AV (2019) The surgical anatomy of the intrapelvic anastomoses of the inferior gluteal artery. Probl Zdorovya Ekologii 60:74–79

[10] Kuzmenko AV, Shkvarko MG (2020) Surgical anatomy of intrapelvic anastomoses of superior gluteal artery. Khirurgiia (11):74–78. 10.17116/hirurgia202011174 33210511

[11] Rodriguez-Niedenfuhr M, Burton GJ, Deu J, Sanudo JR (2001) Development of the arterial pattern in the upper limb of staged human embryos: normal development and anatomic variations. J Anat 199:407–417. 10.1046/j.1469-7580.2001.19940407.x PMC1468351 11693301

[12] Rusu MC, Stancioiu IE, Stancioiu IM, Jianu AM (2026) The mineralised epiglottis: a narrative review illustrated by an index case. Surg Radiol Anat 48:192. 10.1007/s00276-026-03959-y PMC13461744 42581149

[13] Senior HD (1919) The development of the arteries of the human lower extremity. Am J Anat 25:54–95. 10.1002/aja.1000250105

[14] Tunstall R (2016) Internal iliac arteries. In: Tubbs RS, Shoja MM, Loukas M (eds) Bergman’s comprehensive encyclopedia of human anatomic variation. John Wiley & Sons, Hoboken, NJ, pp 694–741

